# Extracorporeal Shock Wave Therapy Alters the Expression of Fibrosis-Related Molecules in Fibroblast Derived from Human Hypertrophic Scar

**DOI:** 10.3390/ijms19010124

**Published:** 2018-01-02

**Authors:** Hui Song Cui, A Ram Hong, June-Bum Kim, Joo Hyang Yu, Yoon Soo Cho, So Young Joo, Cheong Hoon Seo

**Affiliations:** 1 Burn Institute, Hangang Sacred Heart Hospital, College of Medicine, Hallym University, Seoul 07247, Korea; bioeast@hanmail.net (H.S.C.); bsnunki@naver.com (A.R.H.); koko6394@naver.com (J.H.Y.); 2 Department of Pediatrics, Hallym University Hangang Sacred Heart Hospital, Seoul 07247, Korea; hoppdoctor@hanmail.net; 3 Department of Rehabilitation Medicine, Hangang Sacred Heart Hospital, College of Medicine, Hallym University, Seoul 07247, Korea; hamays@hanmail.net

**Keywords:** extracorporeal shock wave therapy, burn hypertrophic scar, hypertrophic scar-derived fibroblast, epithelial-mesenchymal transition, inhibitor of DNA binding protein

## Abstract

Extracorporeal shock wave therapy (ESWT) considerably improves the appearance and symptoms of post-burn hypertrophic scars (HTS). However, the mechanism underlying the observed beneficial effects is not well understood. The objective of this study was to elucidate the mechanism underlying changes in cellular and molecular biology that is induced by ESWT of fibroblasts derived from scar tissue (HTSFs). We cultured primary dermal fibroblasts derived from human HTS and exposed these cells to 1000 impulses of 0.03, 0.1, and 0.3 mJ/mm^2^. At 24 h and 72 h after treatment, real-time PCR and western blotting were used to detect mRNA and protein expression, respectively, and cell viability and mobility were assessed. While HTSF viability was not affected, migration was decreased by ESWT. Transforming growth factor beta 1 (TGF-β1) expression was reduced and alpha smooth muscle actin (α-SMA), collagen-I, fibronectin, and twist-1 were reduced significantly after ESWT. Expression of E-cadherin was increased, while that of N-cadherin was reduced. Expression of inhibitor of DNA binding 1 and 2 was increased. In conclusion, suppressed epithelial-mesenchymal transition might be responsible for the anti-scarring effect of ESWT, and has potential as a therapeutic target in the management of post-burn scars.

## 1. Introduction

Post-burn hypertrophic scars (HTSs) are a most common complication of burn injury and result from excessive scar tissue formation in prolonged aberrant wound healing, and are characterized by hyalinized collagen bundles. They can functionally (arthrentasis), symptomatically (pruritis, pain), and aesthetically impact the patient’s quality of life. Depending on the depth of the wound, hypertrophic scar (HTS) occurs in up to 91% of burn injuries [1]. The pathophysiology of HTS formation involves an overactive proliferative phase in wound healing [2]. A variety of cells (macrophages, fibroblasts, keratinocytes), cytokines, and growth factors participate this process. Activated T cells, macrophages, and Langerhans cells disrupt normal wound healing and tissue remodeling, and contribute to abnormal extracellular matrix (ECM) accumulation with an increased cellular activity [3,4].

In particular, transforming growth factor beta 1 (TGF-β1) has potent stimulatory effects on ECM protein synthesis, and fibroblast proliferation and differentiation [3]. A previous clinical study revealed that the serum levels of TGF-β in patients with burn injuries are approximately twice of those in control patients [5]. Furthermore, TGF-β1 and 2 receptors are overexpressed in fibroblasts of HTS tissues [6]. 

There is clear evidence of elevated fibrotic markers, such as collagen type I, alpha smooth muscle actin (α-SMA), vimentin, fibroblast specific protein 1 (FSP-1), and N-cadherin, and a loss of E-cadherin in human HTS, but not in fibroblasts derived from scar tissue (HTSFs) [7]. The mRNA and protein expression of TGF-β1 is significantly increased in HTSF as compared to normal cells [8]; and the numbers of total fibroblasts and αSMA-positive myofibroblasts are significantly higher in HTS than in normotrophic scar or normal skin [9]. Myofibroblasts are fibroblasts that are activated by TGF-β and other growth factors, and are an important source of cells for the synthesis and secretion of ECM components, which are critical for pathological HTS formation [2]. 

At least three subpopulations have been identified in the dermis: superficial fibroblasts, reticular fibroblasts, and fibroblasts associated with hair follicles; of these, reticular cells originating from the deep dermis contribute to HTS formation [10,11]. At the same time, reticular cells show specific characteristics, including larger cell size, slower proliferation in culture, higher TGF-β1 and collagen production, and higher α-SMA expression when compared with fibroblasts from other dermal layers [10]. However, studies on the pathological characteristics of HTSFs are lacking.

Extracorporeal shockwave therapy (ESWT) is a non-invasive physiotherapy that was first used in the lithotripsy of kidney stones [12]. With its development, it has been gradually applied in the treatment of musculoskeletal diseases, such as plantar fasciitis and chronic lateral epicondylitis (tennis elbow), for which it is approved by the US Food and Drug Administration [13]. Several experimental studies demonstrated that ESWT induces nitric oxide (NO) production and inhibition of nuclear factor kappa B (NF-κB) activation in an in vitro model [14], and significantly induces the expression of 84 angiogenic genes in normal mice or mice with non-healing diabetic ulcers [15]. Further, ESWT induces angiogenic and proliferative growth factors, such as nitric oxide synthase (eNOS), vascular endothelial growth factor (VEGF), and proliferating cell nuclear antigen (PCNA), at joints to stimulate the formation of new capillaries and muscularized vessels, thus improving blood supply and tendon regeneration in animal models [16,17]. Furthermore, clinical trials have shown encouraging, significant correlations of ESWT, with an improved healing rate and complete epithelialization, depending on wound size [18]. One clinical study in patients with post-burn HTSs showed that ESWT softened scars and improved their appearance [19]. Very recently, we reported that ESWT significantly reduced scar pain and pruritus in burn patients during rehabilitation [20,21].

Here, we examined molecular changes induced in HTSFs by ESWT, to reveal the mechanism underlying the beneficial effect of ESWT.

## 2. Results 

### 2.1. Characterization of HTSFs

**Figure 1:**
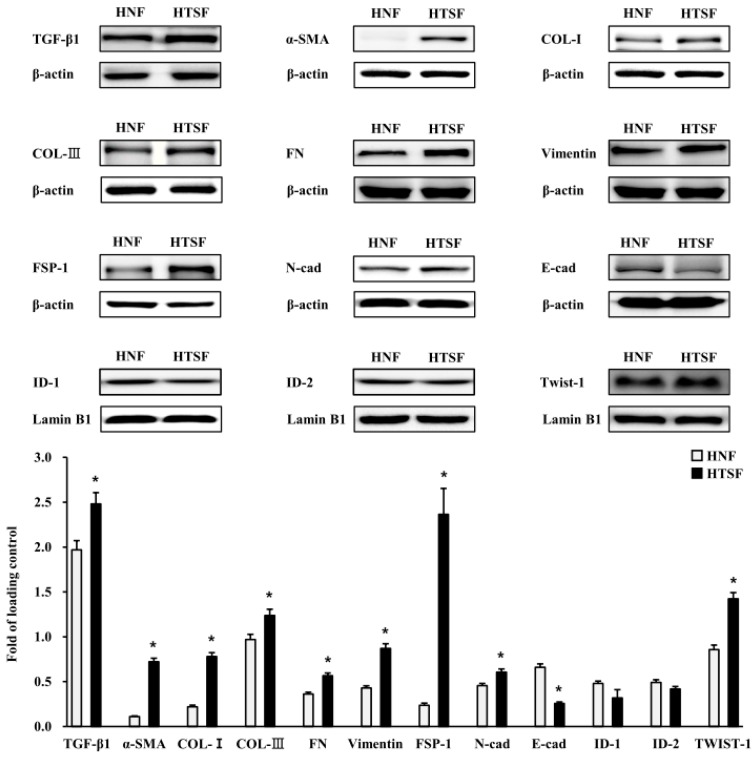
The characteristics of fibroblasts derived from scar tissue (HTSFs). Matched human normal fibroblasts (HNF) and HTSF were cultured from four patients with post burn hypertrophic scar tissue. Protein expression of transforming growth factor beta 1 (TGF-β1), alpha smooth muscle actin (α-SMA), COL-Ι (collagen type Ι), COL-III (collagen type III), FN (fibronectin), Vimentin, fibroblast specific protein 1 (FSP-1), Twist-1 and N-cad (N-cadherin) was significantly higher in HTSFs compared with HNF from skin dermis. The protein expression of E-cad (E-cadherin), inhibitor of DNA binding protein 1 (ID-1) and inhibitor of DNA binding protein 2 (ID-2) were lower in HTSFs when compared with HNF from skin dermis. That expression of those proteins was measured by western blotting against specific antibody. The intensity of band was normalized with that of loading control, β-actin or lamin B1, respectively; HNF, Human normal skin derived fibroblast; HTSF, human hypertrophic scar derived fibroblast. * *p* < 0.05 vs. the corresponding HNF.

To determine the character of HTSFs, we conducted western blot analysis. epithelial-mesenchymal transition (EMT) markers were significantly more strongly expressed by HTSFs than by human normal fibroblasts (HNFs) of the same passage. Protein expression of TGF-β1, α-SMA, collagen-I, collagen-III, fibronectin, vimentin, FSP-1, E-cadherin, and twist1 was significantly higher in HTSFs than in HNFs (Figure 1). In contrast, N-cadherin expression was significantly lower in HTSFs than in HNFs (Figure 1). These results indicated that EMT is closely involved in the pathology of HTS formation.

### 2.2. Effects of ESWT on HTSF Viability

**Figure 2:**
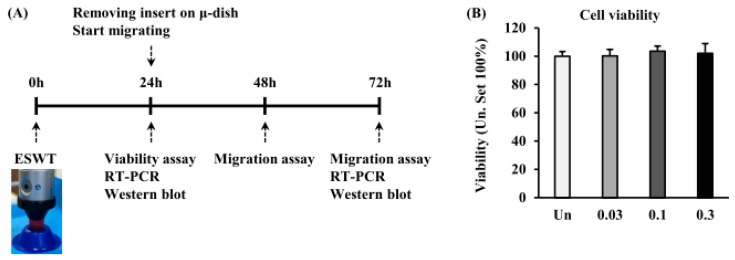
Experimental schematic diagrams and viability of HTSFs. The dermis was separated from human hypertrophic scar tissue by dispase, and then digested with collagenase type IV. HTSF was released, collected, suspended in medium, and continue cultured. After detachment, HTSFs were suspended in to a 17 mL conical tube. ESWT is performed with 1000 impulse/cm^2^ at 0.03, 0.1, and 0.3 mJ/mm^2^ of energy flux densities. Then, HTSFs were seeded on 96 well cell culture plates for viability assays, μ-dish for migration assays, and T75 culture plates for RT-PCR and western blot (0 h). After 24 h, the viability of HTSF was measured. Once removes insert of μ-dish, the HTSF begins to move, and then analyzed after migration 24 and 48 h (48 and 72 h after ESWT). Real time polymerase chain reaction (RT-PCR) and western blot were performed 24 h and 72 h after plating, respectively (**A**). ESWT no influence on viability of HTSFs (**B**). Cell viability was determined using an Cell Titer-Glo^®^ Luminescent cell viability assay kit 24 h after ESWT. Each group was assayed in sextuplicate, and the experiments were performed at three times independently. HTSF viability was expressed as a percentage value of untreated cells. Un: untreated cells.

We investigated the viability of HTSFs after ESWT with 1000 impulses/cm^2^ at 0.03, 0.1, and 0.3 mJ/mm^2^ of energy flux densities at 24 h after plating using a viability assay (Figure 2A). ESWT had no effect on the viability of HTSFs when compared to non-treated cells. ESWT-HTSFs showed a normal growth pattern, with no effect of ESWT on growth rate (Figure 2B). The mRNA levels of glyceraldehyde-3-phosphate dehydrogenase (GAPDH) and β-actin were not affected by ESWT. Furthermore, mRNA expression of bcl-2-associated X protein (bax) and B-cell lymphoma 2 (bcl-2), apoptotic-related factors, was not affected at 24 h and 72 h after ESWT in HTSF (Figures S1 and S2).

### 2.3. Effects of ESWT on TGF-β1, α-SMA and Vimentin Expression in HTSFs

**Figure 3:**
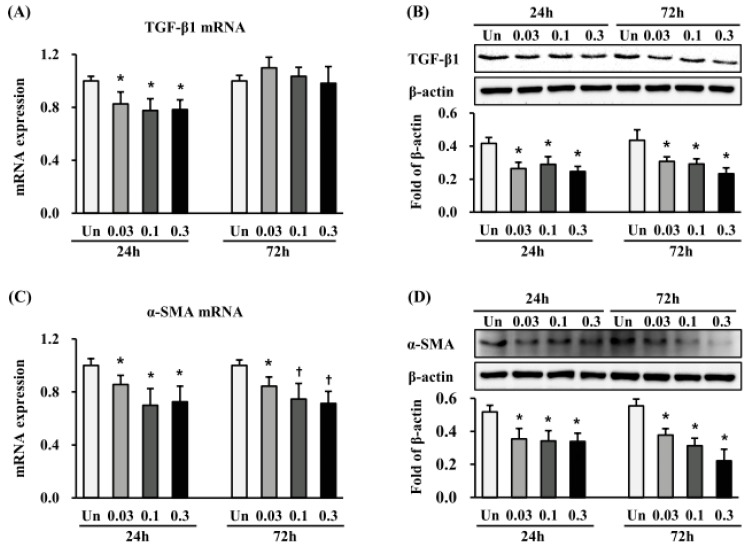
Extracorporeal shockwave therapy (ESWT) decreases the expression of TGF-β1 and alpha smooth muscle actin (α–SMA) in HTSFs. HTSF was cultured from four patients with post burn hypertrophic scar tissue. The mRNA expression of TGF-β1 (**A**) and α–SMA (**C**) were measured 24 h and 72 h after ESWT using a Light Cycler real-time PCR system. Each sample was assayed in duplicate, and experiments were performed least three times independently. The mRNA expression was normalized as ratio = 2^−∆∆*Ct*^, and data are the mean ± S.E. * *p* < 0.05 and † *p* < 0.01 vs. the corresponding untreated control group. Protein expression of TGF-β1 (**B**) and α–SMA (**D**) were measured with western blot analysis 24 and 72 h after ESWT, respectively. The protein expression was normalized with β-actin, respectively; and data are the mean ± S.E. * *p* < 0.05 vs. the corresponding untreated control group. Un: Untreated cells.

As TGF-β1 plays a critical role as a potent EMT inducer in the pathogenesis of HTS formation, we first investigated whether the anti-scarring effect of ESWT was partially mediated via suppression of TGF-β1 expression. The TGF-β1 mRNA level was significantly reduced 24 h after ESWT with 1000 impulses/cm^2^ at 0.03, 0.1, and 0.3 mJ/mm^2^, when compared with non-treated cells (*p* < 0.05) (Figure 3A), while no further changes were observed at 72 h after ESWT. TGF-β1 protein expression was also significantly decreased 24 h and 72 h after ESWT with 1000 impulses/cm^2^ at all tested energy flux densities, as compared to non-treated cells (*p* < 0.05) (Figure 3C). Next, we investigated the expression of α-SMA, an EMT marker directly regulated by TGF-β1. α-SMA mRNA expression was reduced at 24 h and 72 h after ESWT with 1000 impulses/cm^2^ at 0.03, 0.1, and 0.3 mJ/mm^2^, compared with non-treated cells (*p* < 0.05 or 0.01) (Figure 3B). Similar to TGF-β1 protein expression, α-SMA protein expression was significantly decreased at 24 h and 72 h after all ESWT regimens, as compared with non-treated cells (*p* < 0.05) (Figure 3D). Finally, we measured the expression of vimentin, another EMT marker. Vimentin expression was also significantly decreased at 24 h and 72 h after ESWT, when compared with non-treated cells (*p* < 0.05) (Figure S3). On the other hand, non-treated cells were larger and expressed higher levels of vimentin in the cell body. After ESWT, depending on the energy flux density, the HTSFs exhibited spindle- or stellate-shaped fibroblast-like appearance (Figure S3A,B).

### 2.4. Effects of ESWT on Expression of ECM-Related Proteins in HTSFs

**Figure 4:**
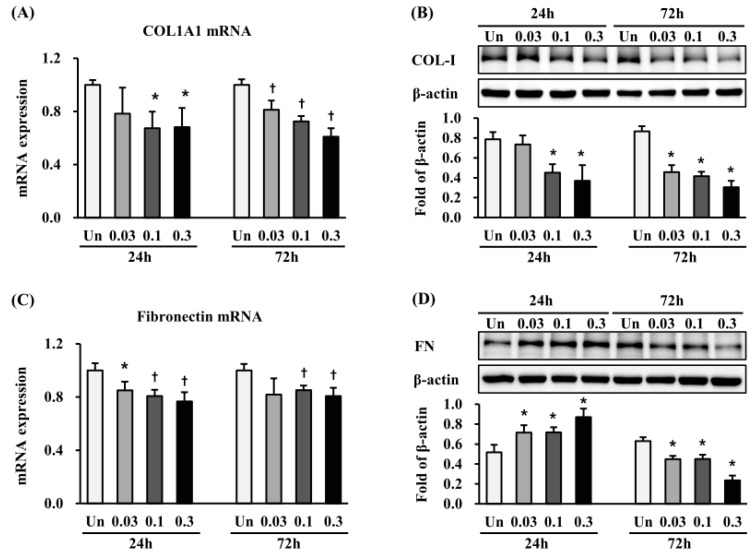
ESWT decreases the expression of extracellular matrix protein in HTSFs. HTSF was cultured from four patients with post burn hypertrophic scar tissue. The mRNA expression of collagen-I (**A**) and fibronectin (**B**) were measured 24 and 72 h after ESWT using a Light Cycler real-time PCR system. Each sample was assayed in duplicate, and experiments were performed least three times independently. The mRNA expression was normalized as ratio = 2^−∆∆*Ct*^, and data are the mean ± S.E. * *p* < 0.05 and † *p* < 0.01 vs. the corresponding untreated control group. Protein expression of collagen-Ι (**C**) and fibronectin (**D**) were measured with western blot analysis 24 and 72 h after ESWT, respectively. The protein expression was normalized with β-actin, respectively; and data are the mean ± SE. * *p* < 0.05 vs. the corresponding untreated control group. Un: Untreated cells.

We investigated the expression of Collagen1a1 and fibronectin, both markers of EMT, at 24 h and 72 h after ESWT. Collagen1a1 mRNA expression was significantly decreased at 24 h after ESWT with 1000 impulses/cm^2^, at 0.03 and 0.1 mJ/mm^2^ and at 72 h after all the ESWT regimens, compared with non-treated cells (*p* < 0.05 or 0.01) (Figure 4A). Accordingly, collagen-I protein expression was significantly reduced at 24 h and 72 h after ESWT (*p* < 0.05) (Figure 4C). Fibronectin mRNA expression was significantly decreased at 24 h after ESWT with 1000 impulses/cm^2^, at 0.03, 0.1 and 0.3 mJ/mm^2^, and at 72 h after ESWT with 1000 impulses/cm^2^, at 0.03, and 0.1 mJ/mm^2^, when compared with non-treated cells (*p* < 0.05 or 0.01) (Figure 4B). Surprisingly, fibronectin protein was significantly increased 24 h after ESWT with 1000 impulses/cm^2^ at 0.03, 0.1, and 0.3 mJ/mm^2^ (*p* < 0.05) (Figure 4D), but significantly reduced at 72 h after ESWT under the same conditions (*p* < 0.05) (Figure 4D).

### 2.5. Effects of ESWT on Expression of N- and E-Cadherin in HTSFs

**Figure 5:**
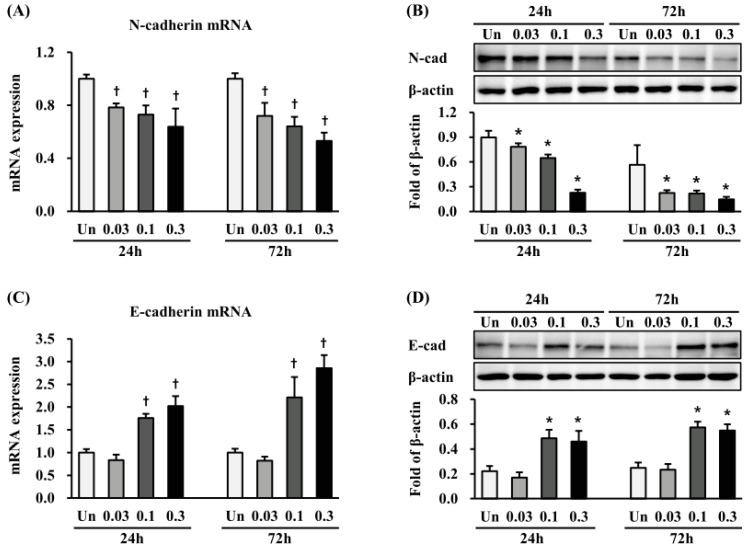
Effects of ESWT on the expression of N-cadherin and E-cadherin in HTSFs. HTSFs were cultured from four patients with post burn hypertrophic scar tissue. The ESWT decreased the mRNA expression of N-cadherin (**A**), and increased the mRNA expression of E-cadherin (**B**). The mRNA expression was measured 24 h and 72 h after ESWT using a Light Cycler real-time PCR system. Each sample was assayed in duplicate, and experiments were performed least three times independently. The mRNA expression was normalized as ratio = 2^−∆∆*Ct*^, and data are the mean ± S.E. * *p* < 0.05 and † *p* < 0.01 vs. the corresponding untreated control group. ESWT decreased the protein expression of N-cadherin (**C**), and increased protein expression of E-cadherin (**D**). Protein expression of N-cadherin and E-cadherin were measured with western blot analysis 24 h and 72 h after ESWT, respectively. The protein expression was normalized with β-actin, respectively, and data are the mean ± S.E. * *p* < 0.05 vs. the corresponding untreated control group. Un: Untreated cells.

We measured the expression of N-cadherin and E-cadherin, which are cell-surface markers of EMT. mRNA expression of N-cadherin was significantly decreased at 24 h and 72 h after ESWT in all the regimens (*p* < 0.05 or 0.01) (Figure 5A), while that of E-cadherin was significantly increased 24 h and 72 h after ESWT with 1000 impulse/cm^2^ at 0.1 and 0.3 mJ/mm^2^ energy flux densities, respectively (*p* < 0.01) (Figure 5B). Similar to mRNA expression, at 24 and 72 h after ESWT in all the regimens, N-cadherin was significantly decreased (*p* < 0.05) (Figure 5C), while E-cadherin was significantly increased 24 h and 72 h after treated with 1000 impulse/cm^2^ at 0.1 and 0.3 mJ/mm^2^, respectively (*p* < 0.05) (Figure 5D).

### 2.6. Effects of ESWT on Transcription Factor Expression in HTSFs

**Figure 6:**
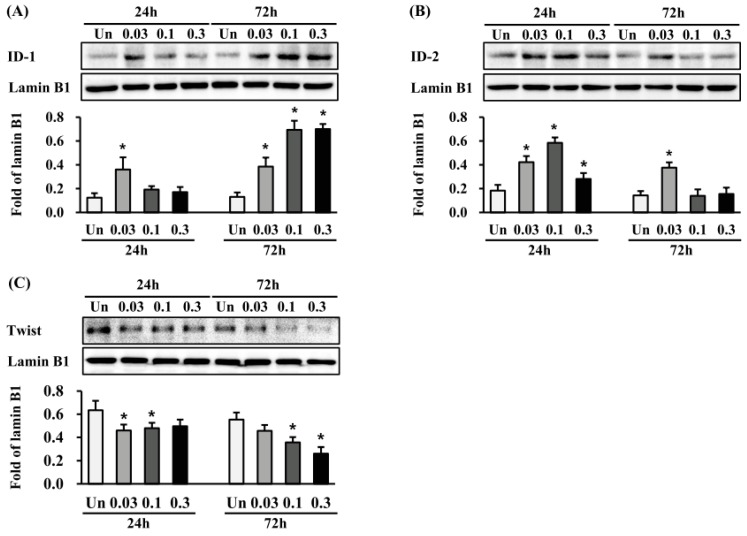
ESWT regulates the expression of ID-1, ID-2, and twist-1 in HTSFs. HTSF was cultured from four patients with post burn hypertrophic scar tissue. The Protein expression of ID-1 (**A**), ID-2 (**B**) and (**C**) were measured with western blot analysis 24 h and 72 h after ESWT, respectively. The protein expression was normalized with lamin B1, and data are the mean ± S.E. * *p* < 0.05 vs. the corresponding untreated control group. Un: Untreated cells.

We investigated whether ESWT regulates the expression of the transcription factors inhibitor of DNA binding 1 (ID-1) and inhibitor of DNA binding 2 (ID-2), both of which are known to be involved in anti-fibrotic effects [22,23]. ID-1 protein expression was significantly induced at 24 h after ESWT with 1000 impulses/cm^2^ at 0.03 mJ/mm^2^ and at 72 h after 1000 impulses/cm^2^ at 0.03, 0.1, and 0.3 mJ/mm^2^ (*p* < 0.05) (Figure 6A). In contrast, ID-2 protein expression was significantly induced at 24 h after ESWT with 1000 impulses/cm^2^ at 0.03, 0.1 and 0.3 mJ/mm^2^, and at 72 h after 1000 impulses/cm^2^ at 0.03 mJ/mm^2^ (*p* < 0.05) (Figure 6B). Furthermore, twist-1 protein expression was significantly reduced at 24 and 72 h after ESWT, respectively (*p* < 0.05) (Figure 6C). These results suggested that expression of transcription factors ID-1, ID-2, and twit-1 might be implicated in the anti-scarring effect of ESWT.

### 2.7. Effects of ESWT on HTSF Migration

**Figure 7:**
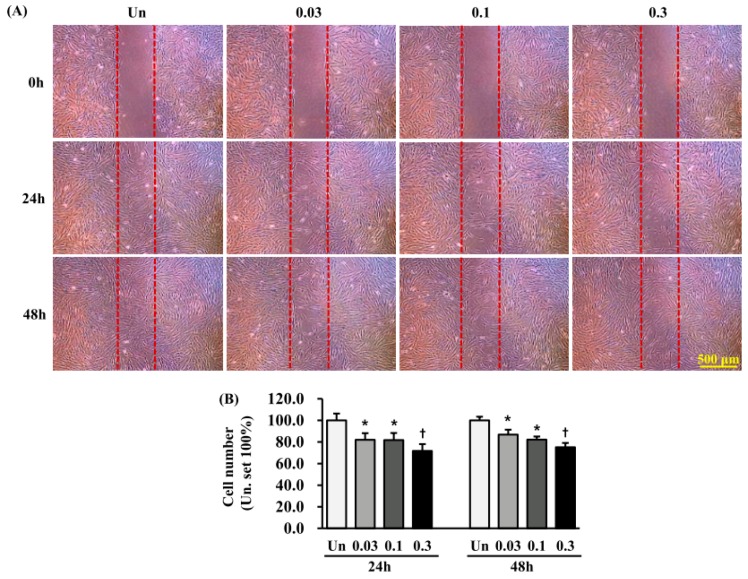
ESWT decreases migration of HTSFs. HTSFs were cultured from four patients with post burn hypertrophic scar tissue. (**A**) The HTSF cells seeded in a culture insert in a 35 mm µ-dish after ESWT, and then after 24 h, the culture insert was removed, made a cell-free gap, allow cells to migrate for 24 h and 48 h. The images were photographed under a light microscopy (scale bar, 500 µm). (**B**) Quantitative analysis of the migration assay was expressed as a percentage relative to untreated cells. The untreated cells were used as control, set to 100%. Data are the mean ± S.E. * *p* < 0.05 and † *p* < 0.01 vs. the corresponding untreated control group. Un: Untreated cells.

For migration assays, HTSFs were seeded in an insert culture system after ESWT, because when ESWT was applied to cells cultured on a dish, the cells detached from the dish. HTSF migration was significantly reduced 24 h (48 h after ESWT) and 48 h (72 h after ESWT) after transfer to the insert upon ESWT with 1000 impulses/cm^2^ at 0.03 and 0.1 mJ/mm^2^, when compared with non-treated cells (*p* < 0.05) (Figure 7). Even more strongly reduced migration was observed when the energy flux density was 0.3 mJ/mm^2^ (*p* < 0.01) (Figure 6).

## 3. Discussion

Previous studies have well documented the clinical effectiveness of ESWT in burn scars [19,20,21]. However, the molecular mechanism remained poorly understood. In the present study, we investigated changes in cell biological behaviors to elucidate the therapeutic mechanism of ESWT underlying its anti-scarring effects. The experimental results suggested that unchanged viability, reduced migration, and the suppressed expression of typical EMT makers in HTFS might be involved in the ESWT anti-scar effect.

Recent evidence suggests that EMT, the reverse of MET, is a process that is essential to wound healing that plays a role in fibrogenesis during HTS formation [6,24,25]. EMT is a process by which epithelial cells lose their epithelial cell characteristics and develop properties typical of mesenchymal cells [26]. During EMT, epithelial cells lose cell–cell connections and downregulate epithelial markers, such as E-cadherin, while they upregulate mesenchymal markers, such as α-SMA, N-cadherin, and fibronectin, and display increased migratory activity [25].

We used HTSFs, which are considered “abnormal” cells, as a research model because fibroblasts have extremely heterogeneous multifunctional potency, and as deep dermal fibroblasts have been suggested to play an important role in HTS formation in deep dermis burn injury [11]. Furthermore, HTSFs have an EMT-like phenotype as compared with matched normal fibroblasts. For example, HTSFs reportedly have a myofibroblast-like character [27]; high levels of TGF-β1 and its receptors; elevated expression of growth factors and inflammatory cytokines, such as CTGF, IL-6, and IL-8; and, increased ECM components as fibronectin and collagen [28]. We observed similar characters in our study, suggesting that HTSFs are a suitable model for studying HTS pathology in vitro. However, interestingly, in our results, the HTSF expressed various mesenchymal properties, such as increased expression of EMT markers, including TGF-β1, α-SMA, collagen-I, collagen-III, fibronectin, vimentin, FSP-1, E-cadherin, and twist1, and decreased expression of N-cadherin. This suggests that the characteristics of HTSF should be further studied.

In the current literature on the use of ESWT, not only were different parameters used in each study, but there is also no agreement on how to obtain maximum potential. In in vitro studies, ESWT has been used with 500–1500 pulses at 0.03 mJ/mm^2^ in human umbilical vein endothelial cells [14], 1000 impulses at 0.14 mJ/ mm^2^ in human tenocytes [29], and 0.06–0.50 mJ/mm^2^ in human osteoblasts [30]. In in vivo studies, ESWT has been used with 1000 impulses at 0.18 mJ/ mm^2^ on tendon bone in dogs [16], 500 impulses at 0.12 mJ/ mm^2^ on tendon bone in rabbits [17], and 200 impulses at 0.1 mJ/ mm^2^ on wound in rabbit [15]. In clinical applications, there are reports of 100 impulses at 0.05–0.15 mJ/mm^2^ in burn pruritus and pain [20,21], 500 impulses at 0.13 mJ/ mm^2^ in burned hands [31], 500 impulses at 0.15 mJ/mm^2^ in full-thickness burns [32], and 100 impulses at 0.037 mJ/ mm^2^ on burn scars [19]. In our study, we applied 1000 impulses/cm^2^ at 0.03, 0.1, and 0.3 mJ/mm^2^ to HTSFs to determine the molecular therapeutic mechanism, and convincing results were obtained under these experimental conditions. Thus, these experimental conditions can serve as a reference for animal or clinical studies.

Previous studies indicated that HTSFs exhibit resistance to apoptosis, which is one of the causes of scar formation [11,33]. Our results showed an unchanged viability of HTSFs at 24 h after ESWT with 1000 impulses/cm^2^ at 0.03, 0.1, and 0.3 mJ/mm^2^ energy flux densities. In addition to GAPDH and β-actin, the mRNA expression of bax and bcl-2, apoptosis-related factors, was not affected in HTSF by ESWT (Supplementary Figures S1 and S2), suggesting that the anti-scarring effect of ESWT is not related to cell death. 

In the wound-healing process, fibroblasts that migrate from the wound edge into the wound core participate in re-epithelialization and granulation tissue formation through proliferation. In the remodeling phase, fibroblasts differentiate into myofibroblasts, as characterized by high α-SMA expression. These myofibroblasts synthesize and deposit ECM components, which are responsible for granulation tissue contraction and the development of mature scar tissue. At the same time, matrix metalloproteinases (MMPs) and collagenase are involved in functional degradation and remodeling of the ECM [34]. It has been suggested that MMPs induced by interleukin 13 (IL-13) convert inactive TGF-β1 into its active form [35]. In an unbalanced inflammatory reaction or under severe inflammation, over production TGF-β1 induces over activation and over proliferation of fibroblasts, which in turn, leads to excess ECM deposition, eventually resulting in HTS formation [34]. This study showed that the inhibits expression of TGF-β1, α-SMA, and vimentin, both of which are cytoskeletal markers of EMT directly regulated by TGF-β1, are likely involved in the anti-scarring therapeutic effect. TGF-β1 is a potent EMT inducer that plays a critical role in wound healing and pathological development of HTS. In addition, our results show that ESWT distinctly decreased ECM components, including collagen-I and fibronectin. These results indicate that the induction of MET and suppression of EMT might be involved in the anti-scarring effect of ESWT.

Cell migration starts at embryonic development and occurs throughout the life cycle. Generally, for cells to migrate, they must adhere to ECM through interacting with or binding to cell adhesion molecules, such as selectins, integrins, and cadherins. It has become increasingly evident that cadherin is important in cell migration. In particular, expression levels of N- and E-cadherin are closely involved in EMT [36], where the loss of E-cadherin is correlated with an upregulation of N-cadherin [37]. Increased myofibroblast invasion or migration depends on N-cadherin in cancer or wound healing [38]. Moreover, E-cadherin has been shown to be essential for cell migration in epithelial wound healing [39]. Given the correlation between cadherin expression and cell migration, we evaluated whether the changes in the cadherin expression are accompanied by changes in the migration of HTSFs. qRT-PCR and western blotting results showed that at 24 h and 72 h after ESWT, E- and N-cadherin expression increased and decreased, respectively, under all the treatment conditions, except when 0.03 mJ/mm^2^ energy flux density was used. Moreover, we observed that HTSF migration decreased at 24 and 72 h after ESWT. The increased E-cadherin expression and decreased migration, both MET-related processes, also suggests a relevant association between MET and the anti-fibrosis effect of ESWT.

The ID protein family has four family members (ID1-4), which can bind to basic helix–loop–helix (bHLH) transcription factors to form a heterodimer that inhibits transcription factor–DNA binding [40,41]. Thus, the proteins control cell differentiation and proliferation, and can act as oncogene or tumor suppressor [41]. ID proteins can interact with Myo D protein, which is a myogenic bHLH transcription factor, to negatively regulate myogenic differentiation [40]. Previous studies have demonstrated that TGF-β1-induced differentiation of fibroblasts into myofibroblasts is partially dependent on Myo D. Moreover, shRNA-mediated knockdown of Myo D expression in myofibroblasts resulted in the reversion of TGF-β1-induced responses [42]. Particularly, an in vitro study indicated that overexpression of ID-1 suppresses TGF-β1-induced Smad 2/3 signaling, thus decreasing collagen expression in human dermal fibroblasts [22]. ID-2 protein also has broader anti-fibrotic effects; ID2 transgenic mice show resistance to bleomycin-induced pulmonary fibrosis partially through down-regulation twist. Although, mice with alveolar epithelial cell deletion of Twist developed fibrosis after bleomycin [23]. Furthermore, a report indicated soluble ECM peptide downregulates the mRNA expression of ID1 and ID2 on epithelial cells, MCF-10A, while it induced TGF-β signaling [43]. In our study, ESWT significantly upregulated the protein expression of ID-1 and ID-2, while downregulating the protein expression of twist-1. Our results, supported by the above findings, indicate that the anti-scarring effect or induction MET action of ESWT is probably via the regulation of ID and twist-1 protein expression, although we did not detect the expression of the other ID subtype proteins, ID3 and ID4, which is a limitation of the present study. Moreover, additional models, such as three-dimensional (3D) culture models, should provide more physiologically relevant information. While we showed that the transcription factor twist-1 was increased in HTSF, the involvement of other transcription factors in the response of HTSF to ESWT remains unclear. In addition, more importantly, further study is required in keratinocytes from normal skin or HTS, and animal models.

## 4. Materials and Methods

### 4.1. Primary Cell Culture

HNFs used in this study were derived from skin biopsy, while HTSFs were isolated from burn-injured HTS tissues derived from surgical procedures, and the HNFs and HTSFs were matched from four patients. The scars ranged in age from one to two years. The study was approved by the Hallym University Hangang Sacred Heart Hospital Institutional Review Board (2 July 2014, registration number 2014-062). Briefly, skin and scar tissues were cut into small pieces, soaked in dispase II (Gibco, Waltham, MA, USA) solution, and maintained at 4 °C overnight. The next day, the epidermis was separated from the dermis, and the dermis was digested with collagenase type IV solution (500 U/mL) at 37 °C for 30 min (Gibco, Waltham, MA, USA). The samples were inactivated with complete medium (DMEM) containing 10% fetal bovine serum (FBS) and 1% antibiotic-antimycotic containing penicillin, streptomycin, and amphotericin B (Gibco, Waltham, MA, USA), filtered, and centrifuged at 300× *g* for 5 min. The pellet was resuspended in complete medium, followed by culture at 37 °C in 5% CO_2_. HTSFs at passage 2 were used for all of the experiments [44].

### 4.2. ESWT

HTSFs were serum-starved for 24 h with 0.1% FBS and were then removed from the cell culture flask using cell detachment solution, Accutase^®^ (Thermo Fisher Scientific, Waltham, MA, USA). The cells were suspended 17 mL of starvation medium in 4.5-cm-long conical tubes (Thermo Fisher Scientific, Waltham, MA, USA) at 1.0 × 10^5^/mL. ESWT was conducted using a Duolith SD-1^®^ device (StorzMedical, Tägerwilen, Switzerland) with an electromagnetic cylindrical coil source of focused shock wave (Figure 1). Cells were treated with 1000 impulses/cm^2^ at 0.03, 0.1, and 0.3 mJ/mm^2^ of energy flux density, with a frequency of 4 Hz. After ESWT, the HTSFs were maintained for 24 h or 72 h in starvation medium (0.1% FBS, 1% antibiotic-antimycotic) in 96-well cell culture plates, μ-dishes, and T75 culture plates to continue cultivation for further experiments.

### 4.3. Cell Viability Assay

HTSF viability was assessed using the CellTiter-Glo^®^ Luminescent cell viability assay kit (Promega, Madison, WI, USA). After ESWT, cells were seeded at 1.0 × 10^4^/well on cell culture plates (Corning, New York, NY, USA). After 24 h or 72 h of cultivation, 100 μL of Cell Titer-Glo^®^ reagent was added to the medium, mixed well, and incubated for 10 min at room temperature. Luminescence was recorded using a microplate reader (Brea, CA, USA). Viability was calculated as follows: viability (%) = (sample luminescence − background luminescence)/(control sample luminescence − background luminescence) × 100. 

### 4.4. HTSF Migration Assay

Cell migration was analyzed, as described previously [44], using a culture insert in a 35-mm μ-dish (Ibidi, GmbH, Planegg, Germany), according to the manufacturer’s instructions. After ESWT, cells were seeded at 2.0 × 10^4^/insert. After 24 h, the culture insert was removed, thus generating a cell-free gap of 500 ± 50 μm. To eliminate the impact of cell proliferation during migration, mitomycin C (5 μg/mL) (Sigma, St. Louis, MO, USA) was added to the cultures. Cells were imaged at 24 and 48 h under a light microscope (IX 70, Olympus, Tokyo, Japan), and the number of cells that had migrated into the gap was analyzed with Image J software (NIH, Bethesda, MD, USA). ESWT cells were compared with untreated HTSFs, as a control, for which migration was set to 100%. Each analysis was performed in triplicate. 

### 4.5. qRT-PCR

HTSFs were collected 24 or 72 h after re-cultivation. Total RNA was isolated using a ReliaPrep^TM^ RNA Miniprep Systems (Promega), according to the manufacturer’s instructions. RNA concentration was measured using a nano-drop spectrophotometer (BioTek, Winooski, VT, USA), and 2 μg of RNA was used to generate cDNA with a high-capacity cDNA reverse transcription kit (Thermo Fisher Scientific, Waltham, MA, USA). qPCR was performed on a LightCycler 480 system (Roche, Basel, Switzerland) using 50 ng of cDNA, 0.5 µM primers (Table S1), and a PCR premix (Takara, Siga, Japan). The reaction conditions were as follows: initial denaturation at 95 °C for 10 min, amplification by 40 cycles of 95 °C for 10 s, 60 °C for 30 s, and extension at 72 °C for 20 s. Target gene mRNA levels of were normalized to the level of GAPDH using the 2^−△△*Ct*^ method [45]. Each qPCR was performed in duplicate with cDNA from at least three different HTSF cultures.

### 4.6. Western Blotting

HTSFs were harvested 24 or 72 h after re-cultivation. The cells were washed with ice-cold phosphate-buffered saline (PBS), resuspended in ice-cold RIPA buffer (Biosesang, Seongnam, Korea) containing a complete phosphatase inhibitor (Roche, Basel, Switzerland) and protease inhibitor cocktail (Sigma, St. Louis, MO, USA), and agitated for 30 min at 4 °C. The samples were centrifuged for 20 min (15,000× *g*, 4 °C), and the protein concentrations of the supernatants were determined with a BCA kit (Thermo Fisher Scientific, Waltham, MA, USA). The samples were mixed with 5× sample buffer and were heated at 70 °C for 10 min. Then, they were (30 μg protein/well) electrophoresed in 7.5% or 15% sodium dodecyl sulfate polyacrylamide gel (SDS-PAGE) gel and electro-transferred onto polyvinylidene difluoride (PVDF) membranes, pore size 40 or 20 µm, respectively (Merck Millipore, Billerica, MA, USA). The membranes were blocked with 5% bovine serum albumin (BSA) for 1 h at room temperature and then incubated for 16 h with polyclonal rabbit anti-TGFβ1 antibody (1:500, Santa Cruz Biotechnology, CA, USA), polyclonal mouse anti-αSMA antibody (1:500, Abcam, Cambridge, UK), monoclonal rabbit anti-fibronectin (1:2000, Abcam, Cambridge, UK), polyclonal rabbit anti-collagen-I; antibody (1:100, Abcam, Cambridge, UK), monoclonal rabbit anti-collagen-III (1:2000, Abcam, Cambridge, UK), monoclonal rabbit anti-vimentin (1:1000, Abcam, Cambridge, UK), polyclonal rabbit anti-FSP-1 antibody (1:1000, Merck Millipore, Billerica, MA, USA), monoclonal anti E-cadherin (1:1000, Cell Signaling, Danvers, MA, USA), monoclonal mouse anti N-cadherin (1:1000, Thermo Fisher Scientific, Waltham, MA, USA), monoclonal mouse anti-ID1 antibody (1:200, Santa Cruz Biotechnology, Santa Cruz, CA, USA), monoclonal mouse anti-ID2 antibody (1:200, Santa Cruz Biotechnology, Santa Cruz, CA, USA), and anti-β-actin (1:5000, Cell Signaling, Danvers, MA, USA). The membranes were washed three times (10 min/wash) with tris-buffered saline-tween 20 (TBST) buffer, and then incubated with peroxidase-conjugated anti-rabbit IgG (1:5000, Merck Millipore, Billerica, MA, USA) for 2 h at room temperature. They were then washed three times (10 min/wash) and were developed with an ECL detection kit (Thermo Fisher Scientific, Waltham, MA, USA). Images were obtained using a chemiluminescence imaging system (WSE-6100; Atto, Tokyo, Japan). Band densities were determined with CS Analyzer4 software (Atto, Tokyo, Japan), and normalized to β-actin density.

### 4.7. Statistical Analysis

All results are presented as the mean ± SEM. The Mann-Whitney *U* test was used for comparisons between two groups. Statistical analyses were conducted with PASW statistics 18 (SPSS Inc., Chicago, IL, USA), and *p* < 0.05 was considered significant. 

## 5. Conclusions

In present study, we demonstrated that ESWT suppresses EMT in HTSFs by inhibiting the expression of TGF-β1, a potent EMT inducer, as well as that of α-SMA, collagen-Ι, fibronectin, and N-cadherin, and by upregulating E-cadherin. Moreover, ESWT inhibits the migratory ability of HTSF. These molecular changes contribute to the anti-fibrotic effects of ESWT on HTSFs. The findings explain well the beneficial effects of ESWT observed in the clinic.

## Supplementary Materials

The following are available online at www.mdpi.com/1422-0067/19/1/124/s1, Figure S1: mRNA expression of β-actin and GAPDH in HTSFs after ESWT, Figure S2: mRNA expression of apoptotic related factors in HTSFs after ESWT, Figure S3: Immunocytochemical analysis of vimentin expression in HTSFs after ESWT, Table S1: Real-time PCR primer sequences.
