# Supplementary material for: Extracorporeal Shock Wave Therapy Alters the Expression of Fibrosis-Related Molecules in Fibroblast Derived from Human Hypertrophic Scar
Source: Int J Mol Sci. 2018 Jan 2;19(1):124. doi: 10.3390/ijms19010124 (PMC5796073; doi:10.3390/ijms19010124)
Supplement: Supplementary file 1 [file ijms-19-00124-s001.pdf]

Supplementary Materials: Extracorporeal Shock Wave Therapy Alters the Expression of Fibrosis-Related Molecules in Fibroblast Derived from Human Hypertrophic Scar

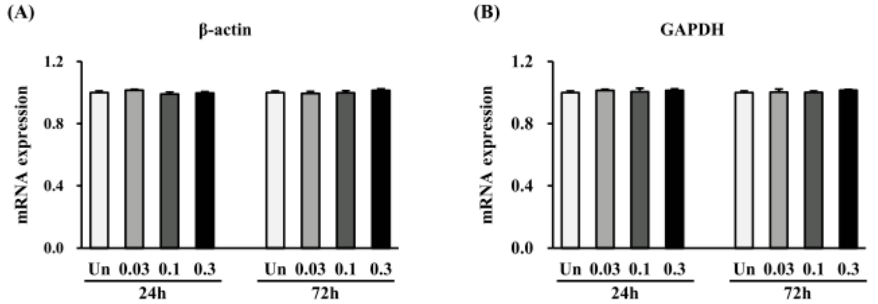

Figure S1. mRNA expression of β-actin and glyceraldehyde-3-phosphate (GAPDH) in hypertrophic scar fibroblasts (HTSFs) after extracorporeal shockwave therapy (ESWT). The mRNA expression of β-actin and GAPDH were not affected by ESWT in HTSF. (**A**) The mRNA expression of GAPDH; (**B**) The mRNA expression of β-actin. Un: Untreated cells.

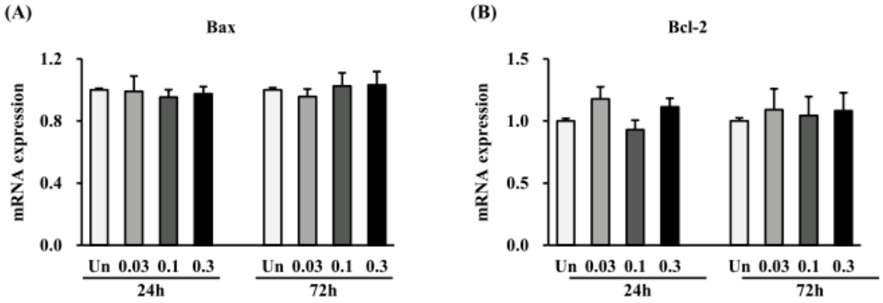

Figure S2. mRNA expression of apoptotic related factors in hypertrophic scar fibroblasts (HTSFs) after extracorporeal shockwave therapy (ESWT). The mRNA expression of bcl-2-associated X protein (bax) and B-cell lymphoma 2 (bcl-2), apoptotic-related factors was not affected by ESWT in HTSF. (**A**) The mRNA expression of bax; (**B**) The mRNA expression of bcl-2. Un: Untreated cells.

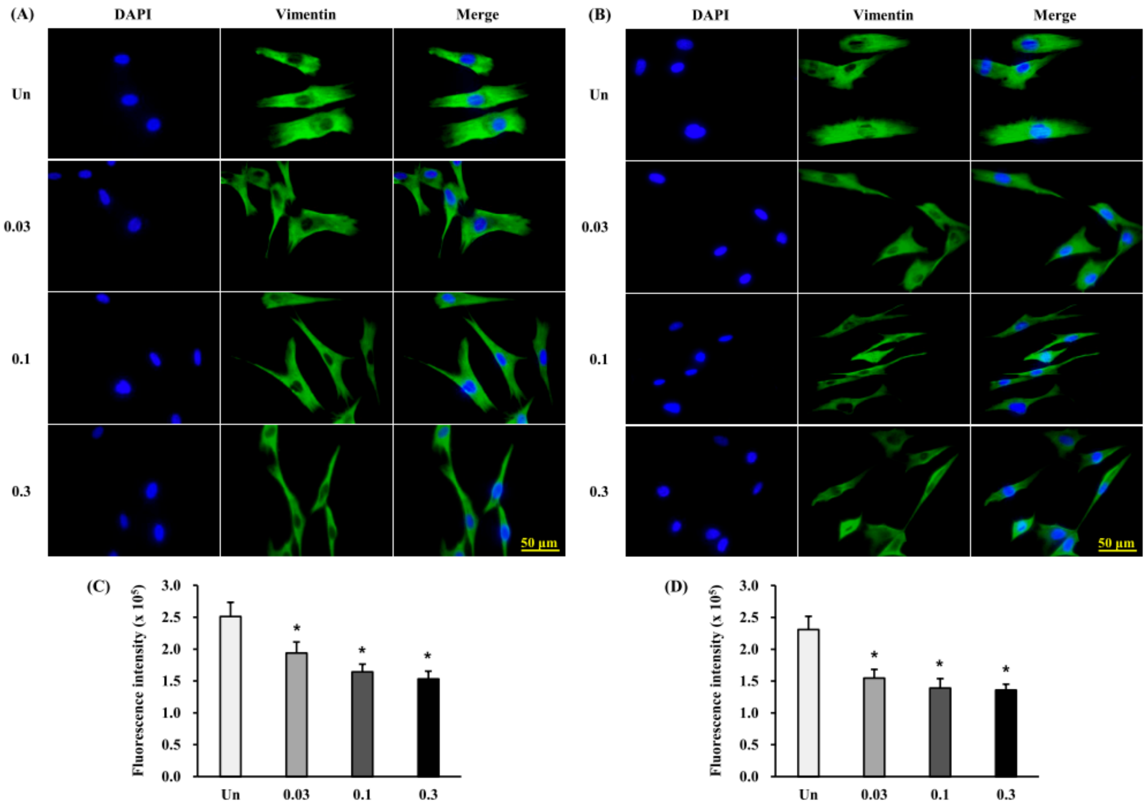

Figure S3. Immunocytochemical analysis of vimentin expression in hypertrophic scar fibroblasts (HTSFs) after extracorporeal shockwave therapy (ESWT). ESWT decreases the expression of vimentin and induces morphological changes of HTSF. The fluorescent immunocytochemistry for vimentin (green) and 4′,6-diamidino-2-phenylindole (DAPI) nuclei (blue) counterstain in HTSFs were performed at 24h and 72h after ESWT, respectively (**A** and **B**). Images were acquired at × 40 magnification, scale bar = 50 μm. Fluorescence intensity quantification of vimentin expression (**C** and **D**). The experiments were performed on three independent HTSF. Data are the mean ± S.E. *$p < 0.05$ the corresponding untreated control group. Un: Untreated cells.

Table S1. Real-time PCR primer sequences.

Gene	Forward (5' → 3')	Reverse (5' → 3')
TGF-β1	CACTCCCGTGGCTTCTAGTG	GTCTTGCAGGTGGAGAGTCC
α-SMA	CCGACCGAATGCAGAAGGA	ACAGAGTATTTGCGCTCCGAA
COL1A1	ATGTTCAGCTTTGTGGACCTC	CTGTACGCAGGTGATTGGTG
Fibronectin	CCAGTCCACAGCTATTCCTG	ACAACCACGGATGAGCTG
E-cadherin	GCAGACCTTCCTCCCAATAC	TGGGTCGTTGTACTGAATGG
N-cadherin	CCACCTTA AAATCTGCAGGC	GTGCATGAAGGACAGCCTCT
Bax	CCTTTTGCTTCAGGGTTTCA	CCATGTTACTGTCCAGTTCG
Bcl-2	TGCGGCCTCTGTTTGATTT	AGGCATGTTGACTTCACTTGT
β-actin	AGAGCTACGAGCTGCCTGAC	AGCACTGTGTTGGCGTACAG
GAPDH	CATGAGAAGTATGACAACAGCCT	AGTCCTTCCACGATACCAAAGT

TGF-β1: Transforming growth factor beta 1; α-SMA: alpha smooth muscle actin; COL1A1: Collagen1A1; Bax: Bcl-2-associated X protein; Bcl-2: B-cell lymphoma 2; GAPDH: Glyceraldehyde-3-phosphate dehydrogenase.
